# Intraosseous epidermoid cyst in the digit of an atopic dog with chronic pododermatitis: case report

**DOI:** 10.29374/2527-2179.bjvm009225

**Published:** 2026-02-10

**Authors:** Cristiane Deon Figueiredo, Daniela Flores Fernandes, Juliana Maciel Cassali Vieira, Renata Bianco Demartini

**Affiliations:** 1 Autonomous Veterinarian, Porto Alegre, RS, Brazil.; 2 Universidade Luterana do Brasil (LBRA), Canoas, RS, Brazil.

**Keywords:** phalanx, bone lesions, melanoma, onychopathies, radiography, falange, lesões ósseas, melanoma, onicopatia, radiografia

## Abstract

Epidermoid cysts are non-neoplastic lesions characterized by a cavity lined by stratified squamous keratinized epithelium and filled with lamellar keratin. While dermal epidermoid cysts are commonly observed in dogs, the intraosseous form is rarely reported. These cysts must be differentiated from other digital lesions involving bone, particularly malignant tumors, as the latter often require more extensive surgical intervention for definitive management. This report describes a case of an intraosseous epidermoid cyst in a dog diagnosed with atopic dermatitis and chronic pododermatitis.

## Resumo

Os cistos epidermoides são lesões não neoplásicas caracterizadas por serem revestidas por tecido epitelial estratificado pavimentoso queratinizado, envolvendo uma cavidade preenchida por queratina lamelar. Embora cistos epidermoides dérmicos ocorram comumente em cães, há poucos relatos da sua ocorrência na forma intraóssea. Estes cistos devem ser diferenciados de outras lesões digitais com envolvimento ósseo, em especial de tumores malignos, pois estes costumam exigir cirurgia mais ampla para o manejo definitivo. Neste trabalho, é relatado um caso de cisto epidermoide intraósseo em um cão diagnosticado com dermatite atópica, o qual apresentava pododermatite crônica.

## Introduction

Intraosseous epidermoid cysts (IECs) are benign cystic lesions that contain a cream-colored, friable, and concentrically laminated material. The etiopathogenesis of IECs is not yet fully understood, and in human medicine, two main theories have been proposed. One suggests a congenital origin, resulting from the entrapment of ectodermal cells during embryonic development, while the other is associated with epidermal implantation following a traumatic or penetrating injury (Yachnin, 1941). It is likely that vertebral and phalangeal cysts in dogs also have different causes (Thompson & Dittmer, 2017).

In humans, the phalanges are most frequently affected, and they have occasionally been reported as a cause of lytic lesions in the distal phalanx of dogs (Wang et al., 2003; Mimura et al., 2019; Vagias et al., 2020). Two cases in dogs have been reported in vertebral bodies (Liu & Dorfman, 1974; Thompson & Dittmer, 2017). The most common clinical signs of phalangeal IECs, as described in the veterinary and human literature, include swelling with pain in the affected phalanx and nail deformity (Yachnin, 1941; Liu & Dorfman, 1974; Wang et al., 2003; Mimura et al., 2019; Vagias et al., 2020).

In dogs, it is important to differentiate this lesion from malignant digital tumors that cause bone lysis, such as squamous cell carcinomas of the nail bed and malignant melanomas (Thompson & Dittmer, 2017). Radiographically, these lesions are characterized by one or more lytic areas surrounded by a sclerotic rim, with or without periosteal bone proliferation. Histopathology is required for a definitive diagnosis of IECs. Histologically, the cysts are lined by well-differentiated, keratinized stratified squamous epithelium and filled with layers of keratinized squames. They are typically supported by a dense fibrous stroma and surrounded by thickened bony trabeculae (Yachnin, 1941; Liu & Dorfman, 1974; Homer et al., 1992; Wang et al., 2003; Thompson & Dittmer, 2017). The treatment options for IECs reported in dogs include digital amputation and isolated phalangeal amputation (Liu & Dorfman, 1974; Homer et al., 1992; Frank et al., 1995).

## Case description

**Figure 1:**
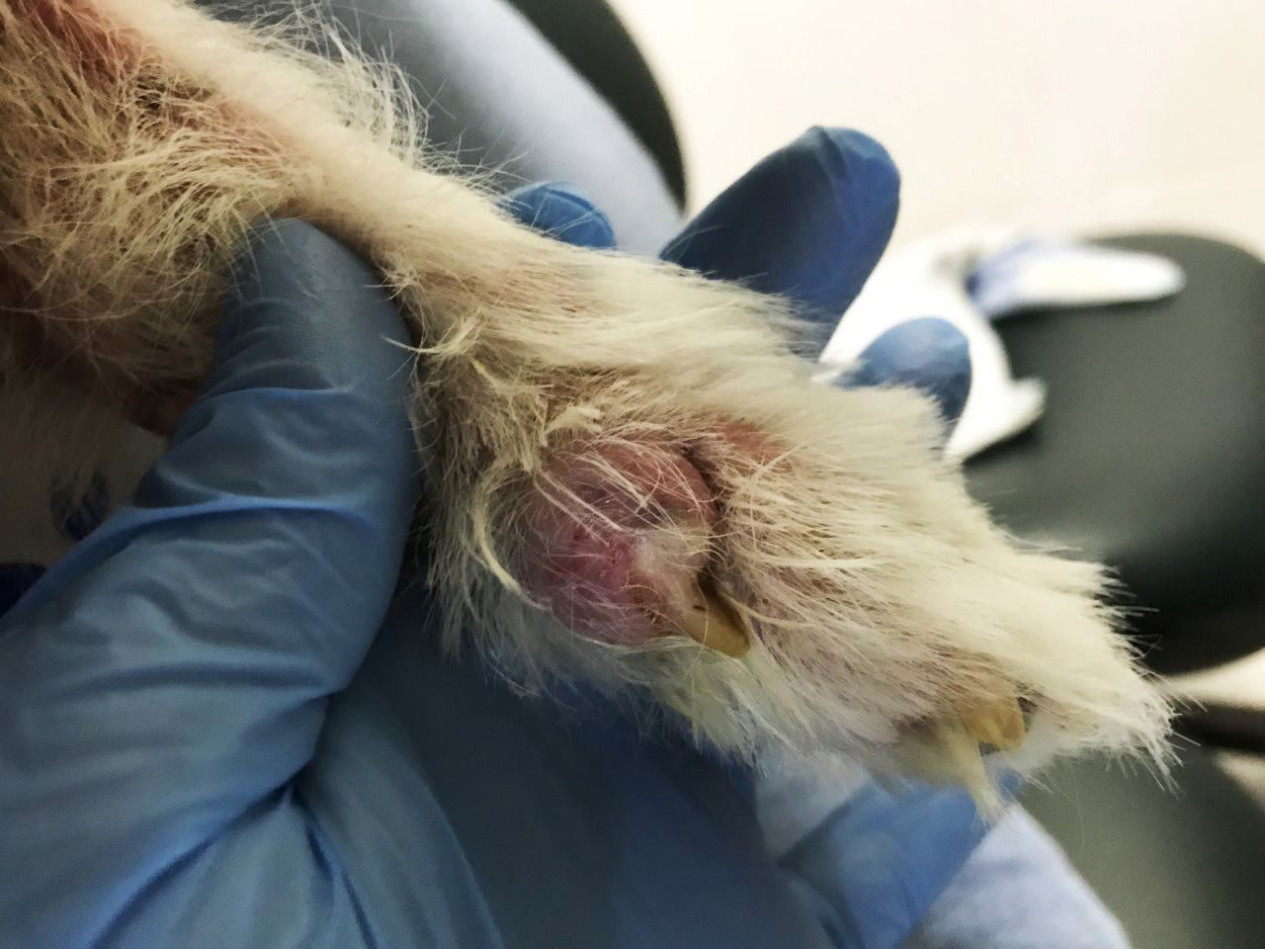
Firm, erythematous, alopecic, and adherent nodule on the fifth digit of the left thoracic limb.

**Figure 2:**
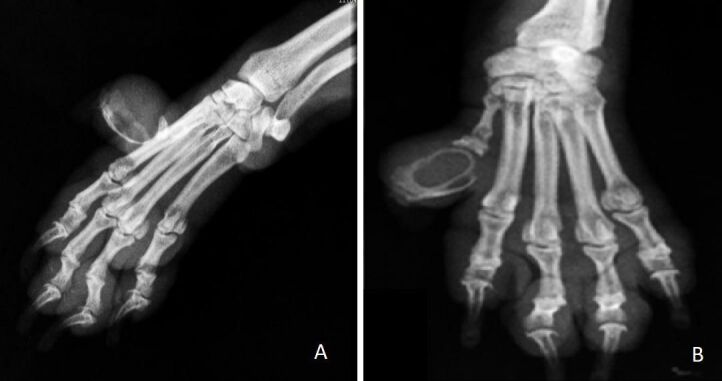
Well-defined osteolysis near the first metacarpal, loss of visualization of the distal phalanx and ungual process, along with increased volume and radiopacity of the adjacent soft tissues of the first digit.

A 9-year-old spayed female Shih Tzu was presented to a dermatology referral service, already under treatment for canine atopic dermatitis (CAD) and recurrent malassezia dermatitis. The owner reported the development of a nodule on one of the digits of the left thoracic limb (Figure 1). Upon examination, the patient had a firm, adherent, erythematous nodule measuring approximately 1.5 cm in the digital region, causing considerable discomfort. Due to the discomfort, a 5-day course of non-steroidal anti-inflammatory therapy was initiated to reduce inflammation and enable fine-needle aspiration cytology (FNAC). No improvement was observed with therapy, and FNAC was performed. The cytological sample revealed a marked number of irregular keratin fragments and a few non-keratinized epithelial cells, cohesively arranged and well-differentiated cytologically. Based on cytology, a diagnosis could not be established; therefore, excisional biopsy of the nodule and subsequent histopathological analysis were recommended. Radiographs were also obtained, which showed marked osteolysis with well-defined margins near the first metacarpal, loss of visibility of the distal phalanx and ungual process, as well as increased volume and radiopacity of the soft tissues adjacent to the first digit (Figure 2a-b), suggesting a differential diagnosis of digital bone neoplasia, bone cyst, or inflammatory process.

**Figure 3:**
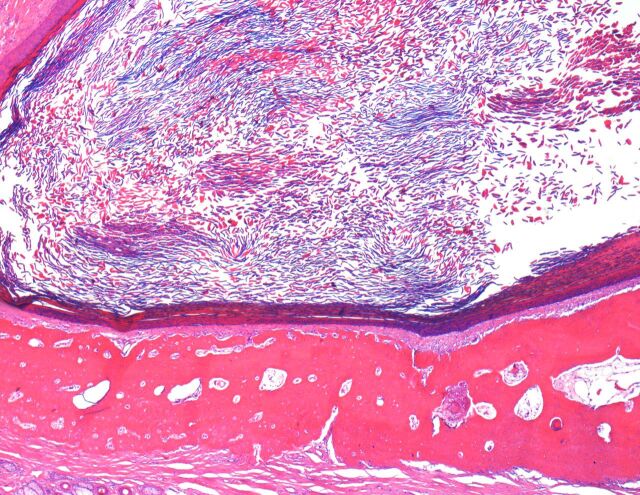
Photomicrograph of a cystic lesion lined by keratinized squamous epithelium and filled with keratin lamellae in the medullary region of the distal phalanx. Obj. 4×, H&E.

Surgical excision of the digit was performed, and the sample was submitted for biopsy. Histopathological examination revealed a cystic structure lined by keratinized stratified squamous epithelium, containing keratohyalin granules on its surface and surrounded by a moderate proliferation of fibrous connective tissue. The structure was filled with lamellar eosinophilic material (keratin) and encased by well-differentiated bone tissue (Figure 3), supporting the diagnosis of an intraosseous epidermoid cyst.

## Discussion

Several alterations, either neoplastic or non-neoplastic, can affect the digits of companion animals. In dogs, one study demonstrated that 53.5% of all digital lesions were aggressive malignant tumors with metastatic potential and a guarded prognosis (Wobeser et al., 2007).

Epidermoid cysts are commonly found in the dermis of dogs and only rarely involve the phalanges (Homer et al., 1992); in such intraosseous cases, the lesion is a non-neoplastic process of unclear origin, although studies in humans suggest a possible embryonic or traumatic origin (Krishnan Unni & Inwards, 2009). Although a correlation is difficult to establish in human medicine, there are several reports of IECs affecting the phalanges of individuals with a history of trauma in the same region (Kumar & Lamba, 2017). In veterinary medicine, only one report has linked cyst development to prior trauma (Vagias et al., 2020). In the present case, the trauma and chronic inflammation caused by excessive and chronic licking of the digits, a clinical sign related to the CAD presented by the animal, may be considered a possible contributing factor to the development of the IEC. The clinical signs observed in this case are similar to those previously described in both dogs and humans, and include swelling with pain in the affected phalanx and nail deformity, consistent with the findings of the present report (Yachnin, 1941; Liu & Dorfman, 1974; Homer et al., 1992; Vagias et al., 2020) .

The lack of response to non-steroidal anti-inflammatory therapy can be explained by the case's etiopathogenesis, as the increase in volume was not due to an inflammatory process. As the radiographic appearance of the lesions was analogous to other digital tumors, presenting as well-defined, expansile radiolucent bone lesions with distinct sclerotic cortical margins and thin walls—as well as due to the similarity of the clinical presentation, excisional biopsy followed by histological analysis was decisive in establishing the definitive diagnosis (Liu & Dorfman, 1974; Fraser et al., 2006; Mimura et al., 2019).

Recurrence of IECs in humans is generally associated with incomplete excision of the cyst wall (Mimura et al., 2019), whereas in dogs, most likely due to digital amputation, no recurrence has been reported (Homer et al., 1992; Vagias et al., 2020). In the present case, amputation of the digit was performed with the initial aim of obtaining tissue for diagnosis; however, it was sufficient as treatment, with no recurrence or development of similar lesions during the four years following surgery up to the time of this report. In addition, the patient received treatment for the control of atopic dermatitis and secondary infections, which is expected to reduce trauma associated with chronic pruritus and consequently decrease the likelihood of recurrence.

## Conclusion

In general, IECs are rare in dogs but should be considered in the differential diagnosis of lesions presenting with digital swelling, particularly in dogs with chronic pododermatitis. Differentiating IECs from other digital lesions with bone involvement, such as malignant tumors, is essential, as these usually require more extensive surgery for definitive management.
